# Rural-urban differences in the initiation of oral anticoagulant therapy in patients with incident atrial fibrillation: A Finnish nationwide cohort study

**DOI:** 10.1371/journal.pone.0276612

**Published:** 2022-10-31

**Authors:** Konsta Teppo, Jussi Jaakkola, Ville L. Langén, Fausto Biancari, Olli Halminen, Miika Linna, Jari Haukka, Jukka Putaala, Pirjo Mustonen, Janne Kinnunen, Alex Luojus, Juha Hartikainen, K. E. Juhani Airaksinen, Mika Lehto

**Affiliations:** 1 University of Turku, Turku, Finland; 2 Heart Unit, Satakunta Central Hospital, Pori, Finland; 3 Division of Medicine, Turku University Hospital, Turku, Finland; 4 Heart and Lung Center, Helsinki University Hospital, Helsinki, Finland; 5 Clinica Montevergine, GVM Care & Research, Mercogliano, Italy; 6 Department of Industrial Engineering and Management, Aalto University, Espoo Finland; 7 Aalto University, Espoo, Finland; 8 University of Eastern Finland, Kuopio, Finland; 9 Faculty of Medicine, Clinicum, University of Helsinki, Helsinki, Finland; 10 Neurology, Helsinki University Hospital and University of Helsinki, Helsinki, Finland; 11 Heart Center, Turku University Hospital, Turku, Finland; 12 Heart Center, Kuopio University Hospital, Kuopio, Finland; 13 Department of Internal Medicine, Lohja Hospital, Lohja, Finland; Brigham and Women’s Hospital, UNITED STATES

## Abstract

**Aims:**

Little is known about rural-urban differences in the treatment and outcomes in patients with atrial fibrillation (AF). We aimed to assess whether the initiation of oral anticoagulant (OAC) therapy in patients with AF differs between those with rural and urban residence.

**Methods:**

The registry-based FinACAF cohort covers all patients with AF from all levels of care in Finland. Patients were divided into rural and urban categories and into urbanization degree tertiles based on their municipality of residence at the time of AF diagnosis. The outcome was the first redeemed OAC prescription.

**Results:**

We identified 222 419 patients (50.1% female; mean age 72.8 (SD 13.2) years) with incident AF during 2007–2018. Urban residence was associated with a lower rate of OAC therapy initiation (adjusted subdistribution hazard ratio (SHR) (95% CI) 0.96 (0.95–0.97)). Correspondingly, an inverse graded dose-response relationship was observed between higher urbanization degree tertile and OAC initiation rate (highest tertile compared to lowest: adjusted SHR (95% CI) 0.94 (0.93–0.95)). The adoption of direct oral anticoagulants for stroke prevention was faster among patients with urban residence.

**Conclusion:**

This nationwide cohort study documented that urban residence is associated with a slightly lower rate of OAC therapy initiation in patients with incident AF, but faster adoption of direct oral anticoagulant use.

## Introduction

Atrial fibrillation (AF), the most common sustained arrhythmia with a prevalence as high as 4.1%, is associated with increased risk of ischemic stroke and mortality [1, 2]. Fortunately, use of oral anticoagulant (OAC) therapy can effectively reduce both the risk of stroke and death in patients with AF and high stroke risk [1, 3]. While vitamin-K antagonists (VKAs) have been the drug of choice for more than 50 years, the emergence of direct oral anticoagulants (DOACs) has revolutionized stroke prevention in AF, and the current guidelines recommend DOACs over VKAs as the first line anticoagulant in non-valvular AF due to their superior safety and efficacy profile [4]. However, underuse of OAC therapy in patients with AF at risk of stroke is common, and considerable disparities in stroke prevention have been reported among patient groups defined by characteristics such as age, race, gender, and mental health status [5–11].

Previous studies have indicated that populations in rural areas have higher all-cause mortality and worse outcomes in cardiovascular diseases [12–14]. Likewise, rural-urban disparities have been reported in the access, utilization, and quality of healthcare [15–18]. However, in patients with AF, studies assessing treatment and outcome differences between rural and urban areas are limited and have shown inconsistent results [19–23]. The objective of this nationwide cohort study covering all patients with AF in Finland was to determine whether the rate of OAC therapy initiation or the choice of DOAC as the initial anticoagulant varies between patients residing in rural and urban areas.

## Methods

### Study population

The Finnish AntiCoagulation in Atrial Fibrillation (FinACAF) Study (ClinicalTrials Identifier: NCT04645537; ENCePP Identifier: EUPAS29845) is a nationwide retrospective cohort study including all patients with an AF diagnosis in Finland during 2004–2018 [2]. Patients were identified from all available national health care registers (hospitalizations and outpatient specialist visits: HILMO; primary health care: AvoHILMO; and National Reimbursement Register upheld by Social Insurance Institute: KELA). The inclusion criterion for the cohort was an International Classification of Diseases, Tenth Revision (ICD-10) diagnosis code I48 (including atrial fibrillation and atrial flutter, together referred as AF) recorded between 2004–2018 and cohort entry occurred on the date of the first recorded AF diagnosis. The exclusion criteria were permanent emigration abroad before December 31^st^ 2018 and age <18 years at AF diagnosis. Owing to the linkage of the national registries and universal coverage of public health insurance in Finland, the data has virtually no loss to follow-up. Follow-up continued until death or 31^st^ December 2018, whichever occurred first. The current substudy was conducted within a cohort of patients with incident AF between 2007 and 2018, established in previous studies of the FinACAF cohort [8, 24]. In this cohort, to include only patients with newly diagnosed AF, a washout period was applied by excluding those with a recorded AF diagnosis during 2004–2006, because the medical history of less than 2-years was considered too short to exclude the presence of a prior AF diagnosis. Additionally, to ensure capturing the true initiation of OAC therapy and exclusion of patients with prior AF, those with a fulfilled OAC prescription during 2004–2006, or within a year before the first AF diagnosis were excluded. Baseline patient characteristics were gathered from medical records from 2004 until cohort entry. Since OAC therapy is not recommended in AF patients with low risk of stroke, sensitivity analyses were performed among patients with at least intermediate stroke risk as well as among patients with high stroke risk (at least intermediate stroke risk: men with CHA_2_DS_2_-VASc score ≥ 1 and women with CHA_2_DS_2_-VASc score ≥ 2; high stroke risk: men with CHA_2_DS_2_-VASc score ≥ 2 and women with CHA_2_DS_2_-VASc score ≥ 3) [4]. The patient selection process is summarized in S1 Fig.

### Rural-urban status

The patients’ were categorized to rural and urban groups according to Finland’s Environmental Administration’s rural-urban classification system and patients’ municipality of residence at cohort entry. In this classification, several variables, such as population, labour, building and road network data, are used to define areas rural-urban status, and urban municipalities have a center with more than 15 000 residents [25]. Additionally, patients were divided into tertiles according to the degree of urbanization of their municipality of residence, acquired from Statistics Finland [26]. The degree of urbanization refers to the proportion of people in a municipality living in localities or urban settlements.

### Initiation of OAC

The primary outcome was the initiation of OAC therapy, which was considered to occur on the date of the first fulfilled OAC (warfarin, apixaban, dabigatran, edoxaban or rivaroxaban) prescription after the cohort entry.

### Study ethics

The study protocol was approved by the Ethics Committee of the Medical Faculty of Helsinki University, Helsinki, Finland (nr. 15/2017) and granted research permission from the Helsinki University Hospital (HUS/46/2018). Respective permissions were obtained from the Finnish register holders (KELA 138/522/2018; THL 2101/5.05.00/2018; Population Register Centre VRK/1291/2019-3; Statistics Finland TK-53-1713-18 / u1281; and Tax Register VH/874/07.01.03/2019). The patients’ identification numbers were pseudonymized, and the research group received individualized, but unidentifiable data. Informed consent was waived due to the retrospective registry nature of the study. The study conforms to the Declaration of Helsinki as revised in 2013.

### Statistical analysis

Statistical analyses were performed with the IBM SPSS Statistics software (version 27.0, SPSS, Inc., Armonk, NY) and R (version 4.0.5, https://www.R-project.org). The chi-square test was used to compare differences between proportions, and the independent samples t-test and analysis of variance to analyze continuous variables. Poisson regression was used to estimate the incidence rates, incidence rate ratios, and adjusted rate differences of OAC initiation. Observation of OAC initiation may be hindered by mortality occurring during study period, and therefore, the Fine-Gray regression with all-cause death as competing event was used to estimate the unadjusted and adjusted subdistribution hazard ratios (SHRs) of OAC initiation. Furthermore, as sensitivity analysis we treated death as informative censoring by estimating stabilized inverse probability of censoring weights for death. These weights were computed by Cox regression according to the following baseline variables: age, gender, calendar year of AF diagnosis, hypertension, heart failure, coronary artery disease, diabetes, prior stroke or transient ischemic attack, abnormal liver function, abnormal kidney function, prior bleeding episodes, dementia, cancer, alcohol use disorder, psychiatric disorders, income, and educational attainment (C statistics for predicting mortality 0.839). Thereafter, we calculated adjusted hazard ratios of OAC initiation with inverse probability weighted Cox regression. Additionally, to determine the factors associated with choosing DOAC over VKA as the initial anticoagulant, binary logistic regression model was used with DOAC initiation as dependent variable including only patients initiating OAC therapy after 2011 when the first DOAC was approved for stroke prevention in patients with AF. The Fine-Gray, Poisson, Cox and binary logistic regression models were adjusted for age, gender, calendar year of AF diagnosis, stroke and bleeding risk factors (hypertension, heart failure, coronary artery disease, diabetes, prior stroke or transient ischemic attack, abnormal liver function, abnormal kidney function, prior bleeding episodes, concomitant use of nonsteroidal anti-inflammatory drugs or antiplatelets), dementia, cancer, alcohol use disorder, psychiatric disorders, income quartiles (according to the maximum personal annual income during 2004–2018) and educational attainment. The definitions of the comorbidities are displayed in S1 Table.

## Results

We identified 222 419 patients (50.1% female, mean age 72.8 (SD13.2) years) with incident AF during 2007–2018. Patients with urban residence had higher educational and income levels, lower prevalence of cardiovascular comorbidities and higher prevalence of psychiatric disorders and alcohol abuse than patients with rural residence (Table 1). The mean follow-up time was 1.3 (SD 2.4) years in patients with rural residence, and 1.4 (SD 2.5) years in patients with urban residence.

**Table 1:** Descriptive characteristics of the cohort.

	Rural-urban status			Urbanization degree tertiles			
	Rural	Urban	P-value	1^st^ (lowest)	2^nd^	3^rd^ (highest)	P-value
	n = 79 567	n = 142 852		n = 74 531	n = 73 704	n = 74 184	
**Demographics**							
Mean age, years	73.4 (12.7)	72.4 (13.5)	<0.001	73.6 (12.6)	72.4 (13.2)	72.2 (13.7)	<0.001
Mean annual income, thousands of euros	17.8 (21.5)	23.9 (24.9)	<0.001	17.3 (21.1)	21.5 (23.1)	26.4 (26.4)	<0.001
Mean degree of urbanization, %	63.4 (14.7)	93.8 (6.5)	<0.001	61.2 (12.8)	88.7 (4.3)	99.0 (1.0)	<0.001
Female sex	39 145 (49.2)	72 254 (50.6)	<0.001	36 872 (49.5)	36 873 (50.0)	37 654 (50.8)	<0.001
**Highest educational level**			<0.001				<0.001
Primary school	46 831 (58.9)	69 063 (48.3)		44 410 (59.6)	37 705 (51.2)	33 779 (45.5)	
Upper secondary school	21 969 (27.6)	38 324 (26.8)		20 499 (27.5)	20 712 (28.1)	19 082 (25.7)	
Higher education	10 767 (13.5)	35 465 (24.8)		9 622 (12.9)	15 287 (20.7)	21 323 (28.7)	
**Income quartiles**			<0.001				<0.001
1^st^ (lowest)	25153 (31.6)	28 665 (20.1)		24 277 (32.6)	16 727 (22.7)	12 814 (17.3)	
2^nd^	20 064 (25.2)	36 303 (25.4)		18 894 (25.4)	19 144 (26.0)	18 329 (24.7)	
3^rd^	18 516 (23.3)	37 875 (26.5)		17 204 (23.1)	19 564 (26.5)	19 623 (26.5)	
4^th^ (highest)	15 834 (19.9)	40 009 (28.0)		14 156 (19.0)	18 269 (24.8)	23 418 (31.6)	
**Comorbidities**							
Abnormal liver function	354 (0.4)	773 (0.5)	0.002	320 (0.4)	365 (0.5)	443 (0.6)	<0.001
Abnormal renal function	3 049 (3.8)	5 883 (4.1)	0.032	2 863 (3.8)	2 886 (3.9)	3 183 (4.3)	<0.001
Alcohol use disorder	2 974 (3.7)	5 886 (4.1)	<0.001	2 856 (3.8)	2 842 (3.9)	3 162 (4.3)	<0.001
Cancer	15 641 (19.7)	30 407 (21.3)	<0.001	14 654 (19.7)	14 870 (20.2)	16 524 (22.3)	<0.001
Coronary artery disease	18 945 (23.8)	31 161 (21.8)	<0.001	17 834 (23.9)	17 096 (23.2)	15 176 (20.5)	<0.001
Dementia	4 100 (5.2)	7 409 (5.2)	0.732	16 855 (22.6)	16 023 (21.7)	15 336 (20.7)	0.003
Diabetes	17 904 (22.5)	30 310 (21.2)	<0.001	10 398 (23.2)	9 588 (21.6)	9 543 (21.8)	<0.001
Dyslipidemia	38 323 (48.2)	68 495 (47.9)	0.328	36 085 (48.4)	36 171 (49.1)	34 562 (46.6)	<0.001
Heart failure	14 451 (18.2)	24 106 (16.9)	<0.001	13 805 (18.5)	12 730 (17.3)	12 022 (16.2)	<0.001
Hypertension	59 824 (75.2)	195 598 (73.9)	<0.001	56 140 (75.3)	54 675 (74.2)	54 610 (73.6)	<0.001
Prior bleeding	8 458 (10.6)	15 503 (10.9)	0.105	7 966 (10.7)	8 023 (10.9)	7 972 (10.7)	0.453
Prior ischemic stroke or TIA	12 712 (16.0)	21 694 (15.2)	<0.001	12 044 (16.2)	11 480 (15.6)	10 882 (14.7)	<0.001
Prior myocardial infarction	7 506 (9.4)	11 871 (8.3)	<0.001	7 082 (9.5)	6 521 (8.8)	5 774 (7.8)	<0.001
Psychiatric disorder	10 140 (12.7)	20 244 (14.2)	<0.001	9 662 (13.0)	10 108 (13.7)	10 614 (14.3)	<0.001
**Risk scores**							
Modified HAS-BLED score	2.5 (1.0)	2.5 (1.1)	<0.001	2.5 (1.0)	2.5 (1.1)	2.5 (1.1)	<0.001
CHA_2_DS_2_-VASc score	3.5 (1.9)	3.4 (1.9)	<0.001	3.5 (1.9)	3.4 (1.9)	3.4 (1.9)	<0.001
Low stroke risk	5 847 (7.3)	12 541 (8.8)	<0.001	5 268 (7.1)	6 373 (8.6)	6 474 (9.1)	<0.001
Intermediate stroke risk	10 645 (13.4)	20 826 (14.6)	<0.001	9 761 (13.1)	10 714 (14.5)	10 996 (14.8)	<0.001
High stroke risk	63 075 (79.3)	109 485 (76.6)	<0.001	59 502 (79.8)	56 617 (76.8)	56 441 (76.1)	<0.001

Values denote n (%) or mean (standard deviation). Abbreviations: CHA_2_DS_2_-VASc, congestive heart failure, hypertension, age ≥75 years, diabetes, history of stroke or TIA, vascular disease, age 65–74 years, sex category (female); modified HAS-BLED score, hypertension, abnormal renal or liver function, prior stroke, bleeding history, age >65 years, alcohol abuse, concomitant antiplatelet/NSAIDs (no labile INR, max score 8); TIA, transient ischemic attack. Low stroke risk: Men with CHA_2_DS_2_-VASc score 0 and women with CHA_2_DS_2_-VASc score ≤ 1; Intermediate stroke risk: Men with CHA_2_DS_2_-VASc score 1 and women with CHA_2_DS_2_-VASc score 2; High stroke risk: Men with CHA_2_DS_2_-VASc score >1 and women with CHA_2_DS_2_-VASc score > 2.

**Fig 1:**
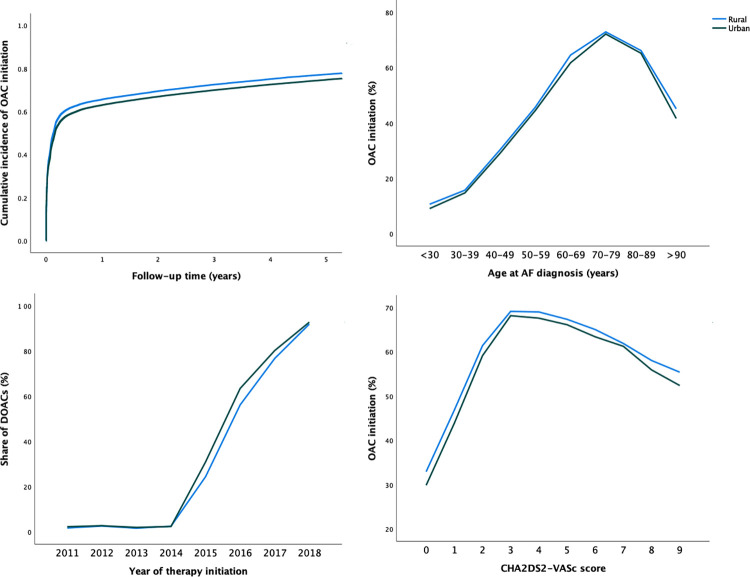
Cumulative incidence curve of OAC initiation (upper left panel), share of DOACs as the initial anticoagulant (lower left panel), and proportions of patients initiating OAC therapy within the first year after AF diagnosis according to age (upper right panel) and CHA_2_DS_2_-VASc score (lower right panel) at AF diagnosis.

OAC therapy was initiated in altogether 72.0% patients with rural residence and 69.9% patients with urban residence. When compared to patients with rural residence, the unadjusted and adjusted rates of OAC initiation were lower among patients with urban residence both in the Fine-Gray and Poisson regression models (Adjusted rate difference -3.4 events/100patients years; Table 2, S3 Table, Fig 1). An inverse dose-response association with higher urbanization degree tertile and OAC initiation rate was also observed (Table 2). An association of similar magnitude was observed in the sensitivity analyses among patients at intermediate to high stroke risk as well as among patients at high stroke risk (S2 Table). Also, the findings were reiterated in the sensitivity analyses considering death as informative censoring (S4 Table).

**Table 2:** Incidence of OAC initiation during follow-up.

	Events	Patient years (1000 years)	Incidence (per patient year)	Unadjusted SHR	Adjusted SHR
**Residence**					
Rural	57 273 (72.0%)	103.1	0.56 (0.55–0.56)	(Reference)	(Reference)
Urban	99 872 (69.9%)*	202.4	0.49 (0.49–0.50)	0.95 (0.94–0.96)	0.96 (0.95–0.97)
**Urbanization degree tertiles**					
1^st^ (lowest)	32 356 (72.2%)	56.7	0.57 (0.56–0.58)	(Reference)	(Reference)
2^nd^	31 825 (71.8%)*	57.3	0.56 (0.55–0.56)	0.98 (0.96–0.99)	0.98 (0.97–0.99)
3^rd^ (highest)	30 795 (70.3%)*	61.8	0.50 (0.49–0.50)	0.91 (0.90–0.93)	0.94 (0.93–0.95)

Abbreviations: SHR, subdistribution hazard ratio. 95% confidence intervals in parenthesis. SHRs estimated by Fine-Gray regression with all-cause death as competing event. Adjusted analyses included the following variables: age, gender, calendar year of AF diagnosis, stroke, and bleeding risk factors (hypertension, heart failure, coronary artery disease, diabetes, prior stroke or transient ischemic attack, abnormal liver function, abnormal kidney function, prior bleeding episodes, concomitant use of nonsteroidal anti-inflammatory drugs or antiplatelets), dementia, cancer, alcohol use disorder, psychiatric disorders, income, and educational attainment.

* = p<0.001.

**Fig 2:**
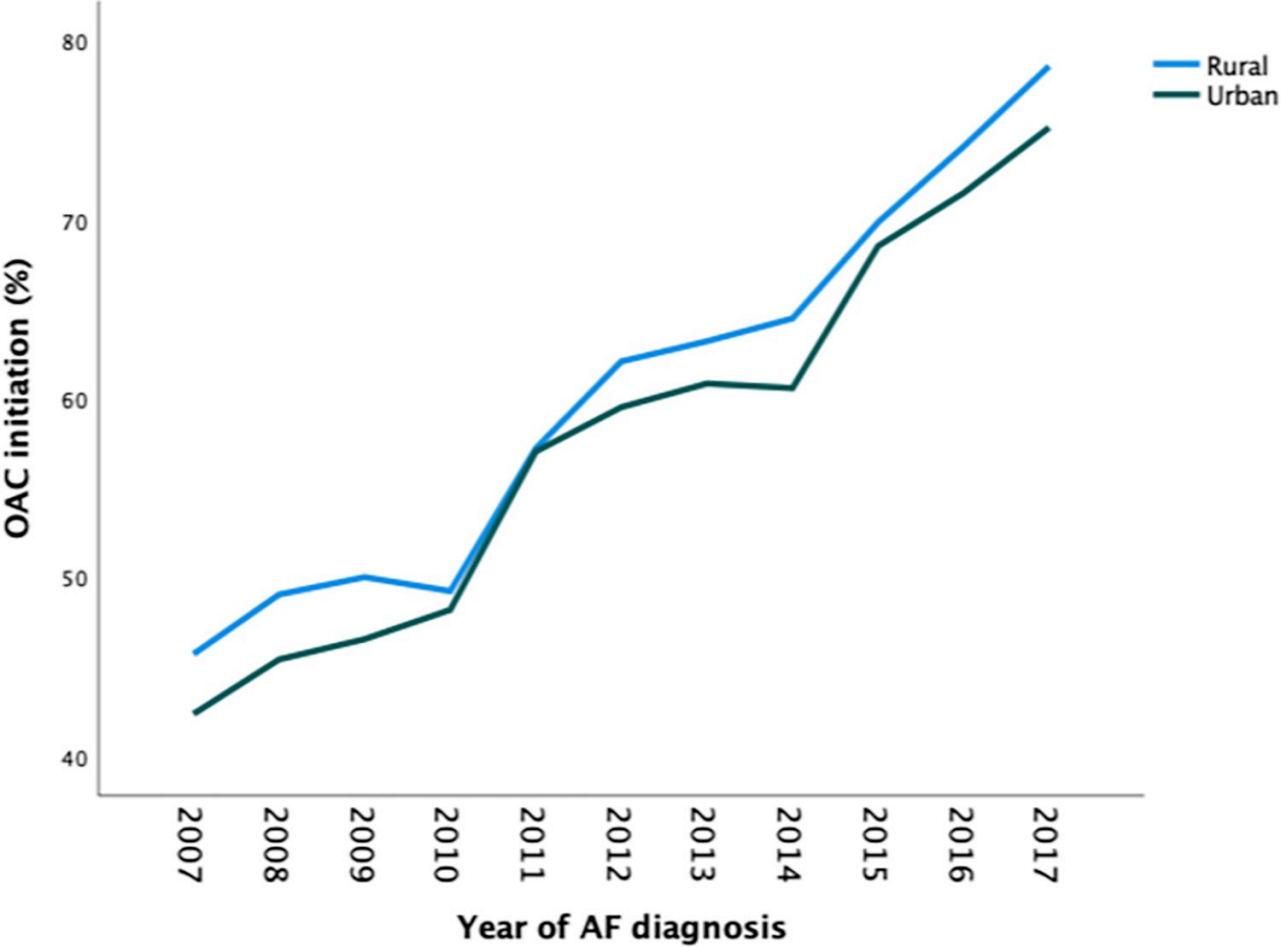
Proportion of patients initiating OAC therapy within the first year after AF diagnosis according to the diagnosis year.

The overall OAC initiation within the first year after AF diagnosis increased continuously between 2007 and 2017 and patients with rural residence were consistently more likely to initiate OAC therapy across the observation period (Fig 2). Patients with rural residence were more likely to initiate OAC within the first year after AF diagnosis in all age groups and across the CHA_2_DS_2_-VASc score scale (Fig 1).

During 2011–2018, OAC therapy was started with a DOAC in 38.5% of patients with rural residence as compared with 41.4% among those with urban residence. A positive association was observed between both urban residence and higher urbanization degree tertile on the likelihood of DOAC as the initial OAC (Fig 1 and Table 3). The share of DOACs as the initial anticoagulant increased rapidly from 2014 onwards, and this increase was faster among patients with urban residence (Fig 1). However, as the share of DOACs as the initial anticoagulant increased, the rural-urban disparity in their use attenuated substantially (OAC initiations during 2018: rural 92.0% vs. urban 92.8%, p = 0.03, Fig 1).

**Table 3:** Proportion of DOAC as the initial anticoagulant of all OAC initiations during 2011–2018 according to rural-urban status.

	DOAC initiations	Unadjusted OR	Adjusted OR
**Residence**			
Rural	17 686 (38.5%)	(Reference)	(Reference)
Urban	33 745 (41.4%)	1.13 (1.10–1.15)	1.18 (1.13–1.22)
**Urbanization degree tertiles**			
1^st^ (lowest)	16 350 (38%)	(Reference)	(Reference)
2^nd^	17 098 (40%)	1.09 (1.06–1.12)	1.08 (1.04–1.12)
3^rd^ (highest)	17 983 (43.2%)	1.24 (1.21–1.28)	1.29 (1.24–1.34)

Abbreviations: DOAC, direct oral anticoagulant; OR, odds ratio. 95% confidence intervals in parenthesis. ORs estimated by binary logistic regression and adjusted for age, gender, calendar year of AF diagnosis, stroke, and bleeding risk factors (hypertension, heart failure, coronary artery disease, diabetes, prior stroke or transient ischemic attack, abnormal liver function, abnormal kidney function, prior bleeding episodes, concomitant use of nonsteroidal anti-inflammatory drugs or antiplatelets), dementia, cancer, alcohol use disorder, psychiatric disorders, income, and educational attainment.

## Discussion

This register-based cohort study covering all patients with incident AF in Finland documented that urban residence is associated with a lower rate of OAC therapy initiation. An inverse dose-response relationship between higher urbanization and OAC therapy initiation was observed. On the other hand, we observed that the broad adoption of DOACs as the initial anticoagulant was considerably faster among patients with urban residence. However, as the use of DOACs increased both in patients with rural and urban residence, by 2018 no clinically meaningful difference in the share of DOACs of all OAC initiations was any longer observed.

Rural-urban disparities in OAC initiation were observed regardless of patients’ age, stroke risk or year of AF diagnosis. Contrary to our expectations, patients with urban residence had a 5% lower unadjusted rate of OAC initiation and urban residence remained independently associated with a lower OAC initiation rate even after adjusting for multiple patient characteristics, including other socioeconomic factors. Similarly, in the sensitivity analyses restricted to high-risk patients, the highest urbanization degree tertile had a 7% lower adjusted OAC initiation rate when compared to the lowest urbanization degree tertile. These results are somewhat discordant with previous observations of higher overall mortality and stroke incidence in rural areas [12]. The magnitude of the observed disparities is relatively small and whether it reflects true clinically significant underuse of OAC therapy in urban areas is unclear. However, especially among the patients at high-risk of ischemic stroke, the observed disparity in stroke prevention may be of clinical significance.

Previous research addressing rural-urban disparities in OAC use in patients with AF is limited and has provided somewhat inconsistent results [19, 21, 22, 27, 28]. Importantly, these studies have been prone to major selection, information, and confounding biases, since they have lacked nationwide study samples, patient data covering all levels of healthcare and individual income and educational levels, and controlling for mortality differences with competing risk models. Therefore, the current study covering all patients with AF in Finland provides substantially more solid evidence and increases our understanding on this topic. Although not all previous studies concur with our observation of lower OAC initiation rate in patients with urban residence, results in concordance with our findings can also be found from studies conducted in the United States and Canada [22, 27, 28] Likewise, our findings of urban residence being associated with a higher likelihood of choosing DOACs over VKA as the initial anticoagulant are in line with previous literature [27, 29].

Factors underlying the observed rural-urban differences in OAC initiation are likely multifactorial. Differences in stroke risk factors and other comorbidities may affect OAC initiation rates between patients with rural and urban residence. However, these factors were controlled for in our adjusted analysis. Additionally, albeit access to healthcare may be worse in rural areas due to longer distances to hospitals, one study reported, in fact, a higher utilization of healthcare in terms of general practitioner visits in rural areas, which may reflect in our findings of higher OAC initiation rate in rural areas [30]. Furthermore, trust between the patient and clinician as well as patients’ preference for lifelong preventive therapies may differ between patients with rural and urban background. On the other hand, the proximity of hospitals and other specialist care may hasten the implementation of novel therapies and guidelines in urban areas and may partly explain the observed faster adoption of DOACs to the mainstream of stroke prevention in patients with urban residence. Additionally, the higher costs of DOACs compared to VKAs may have hindered their use in patients with rural residence owing to their lower income.

The most important limitations of our study are the challenges inherent to register-based retrospective cohort studies, and therefore our observations reflect associations and not necessarily causation. Additionally, we lacked data on the actual patient-level reasons for withholding OAC therapy. Information bias may be present in the used administrative data due to inaccurate recording of data, however, information on OAC initiation is based on a complete nationwide data of claimed prescriptions, including all OAC purchases. Furthermore, while our results rely on pharmacy claims, possible differences in compliance to prescribed OAC therapy may affect our results. Patients with missing data were excluded, but their proportion of the overall cohort was relatively small (3.1%), and this exclusion is unlikely to impact our results significantly. Also, OACs may have been prescribed for indications other than AF during follow-up, although a vast majority of OACs are used for AF [31]. Finally, although the analyses were adjusted for multiple variables, residual confounding by other unmeasured factors cannot also be excluded. Notwithstanding these limitations, the results of this large nationwide cohort study accentuate rural-urban differences in OAC use and emphasize the need for efforts to ensure adequate stroke prevention to all patients with AF. Future research is needed to explore the factors underlying the observed disparities in the utilization of OAC therapy, whether these disparities affect outcomes, as well as possible interventions to improve OAC coverage in patients with AF and urban residence.

In conclusion, this nationwide retrospective cohort study showed that urban residence is associated with a slightly lower rate of OAC therapy initiation, highlighting potential missed opportunities in stroke prevention among patients with AF and urban residence. On the other hand, the broad adoption of DOACs for prevention of AF-related stroke has been faster in urban areas.

## Supporting information

S1 Table Definitions of the comorbidities. (DOCX)

S2 Table Incidence of OAC initiation during follow-up in patients with at least intermediate or high stroke risk. (DOCX)

S3 Table Incidence rate ratios and adjusted rate difference of OAC initiation during follow-up estimated with Poisson regression. (DOCX)

S4 Table Hazard ratios of OAC initiation during follow-up estimated with Cox regression with death considered as informative censoring. (DOCX)

S1 Fig Flow-chart of the patient selection process. (PDF)
