# Supplementary material for: Rural-urban differences in the initiation of oral anticoagulant therapy in patients with incident atrial fibrillation: A Finnish nationwide cohort study
Source: PLoS One. 2022 Oct 31;17(10):e0276612. doi: 10.1371/journal.pone.0276612 (PMC9621410; doi:10.1371/journal.pone.0276612)
Supplement: S1 Table — (DOCX) [file pone.0276612.s001.docx]

**Supplementary Table 1**. Definitions of the comorbidities

|  | ICD-10 | ICPC-2 | Reimbursement code | ATC code | Other |
| --- | --- | --- | --- | --- | --- |
| Hypertension | I10-I15 | K85, K86, K87 | 205 | C03A, C03B, C03DB, C03EA, C07A, C08CA, C08D, C09 |  |
| Dyslipidemia | E78 | T93 | 206 | C10 |  |
| Heart failure | I50, I11.0, I13.0, I13.2 | K77 | 201 |  |  |
| Diabetes | E10-E14 | T89, T90 | 103, 215 | A10 |  |
| Previous stroke | I63, I64, I69.3-I69.8 | K90 |  |  |  |
| Bleeding history | D50.0, D62, D68.3, I60-I62, I69.0-I69.2, I85.0, I86.4, J94.2, K22.1, K22.3, K22.6, K25.0, K25.2, K25.4, K25.6, K26.0, K26.2, K26.4, K26.6, K27.0, K27.2, K27.4, K27.6, K28.0, K28.2, K28.4, K28.6, K29.0, K62.5, K63.1, K63.3, K92.0-K92.2, N02, R04, R31, R58, S06.2-S06.6, S06.8 |  |  |  |  |
| Alcohol abuse | F10 |  |  |  |  |
| Renal failure or dialysis | N18, Z49 |  |  |  |  |
| Liver cirrhosis or failure | K70.2-K70.4, K71.7, K71.8, K72, K74 |  |  |  |  |
| Dementia | F00-F03, G30 |  |  |  |  |
| Cancer |  |  |  |  | Any cancer registered in the Finnish Cancer Registry |
| Coronary heart disease | I21-I25 |  |  |  |  |
| Prior myocardial infarction | I21-I22 |  |  |  |  |
| Psychiatric disorder | F04-F99 |  |  |  |  |

Abbreviations: ATC, anatomic therapeutic chemical; ICD-10, International Classification of Diseases, Tenth Revision; ICPC-2, International Classification of Primary Care, Second Edition
