# Supplementary material for: Rural-urban differences in the initiation of oral anticoagulant therapy in patients with incident atrial fibrillation: A Finnish nationwide cohort study
Source: PLoS One. 2022 Oct 31;17(10):e0276612. doi: 10.1371/journal.pone.0276612 (PMC9621410; doi:10.1371/journal.pone.0276612)
Supplement: S2 Table — (DOCX) [file pone.0276612.s002.docx]

**Supplementary Table 2.** Incidence of OAC initiation during follow-up in patients with at least intermediate or high stroke risk

|  | **Events** | **Patient years (1000 years)** | **Incidence (per patient year)** | **Unadjusted SHR** | **Adjusted SHR** |
| --- | --- | --- | --- | --- | --- |
| **At least intermediate stroke risk (men with CHA_2_DS_2_-VASc score ≥ 1 and women with CHA_2_DS_2_-VASc score ≥ 2)** | | | | | |
| **Residence** | | | | | |
| Rural | 54 555 (74.0%) | 81.3 | 0.67 (0.67-0.68) | (Reference) | (Reference) |
| Urban | 94 524 (72.5%) | 154.5 | 0.61 (0.61-0.62) | 0.96 (0.95-0.97) | 0.96 (0.95-0.97) |
| **Urbanization degree tertiles** | | | | |  |
| 1^st^ (lowest) | 51 241 (74.0%) | 75.2 | 0.68 (0.68-0.69) | (Reference) | (Reference) |
| 2^nd^ | 49 517 (73.4%) | 77.9 | 0.64 (0.63-0.64) | 0.99 (0.98-1.00) | 0.98 (0.97-0.99) |
| 3^rd^ (highest) | 48 321 (71.7%) | 82.7 | 0.58 (0.58-0.59) | 0.93 (0.92-0.94) | 0.93 (0.92-0.95) |
| **High stroke risk (men with CHA_2_DS_2_-VASc score ≥ 2 and women with CHA_2_DS_2_-VASc score ≥ 3)** | | | | | |
| **Residence** |  |  |  |  |  |
| Rural | 47 350 (75.1%) | 56.7 | 0.84 (0.83-0.84) | (Reference) | (Reference) |
| Urban | 81 002 (74.0%) | 104.1 | 0.78 (0.77-0.78) | 0.97 (0.96-0.98) | 0.96 (0.95-0.97) |
| **Urbanization degree tertiles** | | | |  |  |
| 1^st^ (lowest) | 44 620 (75.0%) | 53.0 | 0.84 (0.84-0.85) | (Reference) | (Reference) |
| 2^nd^ | 42 461 (75.0%) | 52.4 | 0.81 (0.80-0.82) | 1.00 (0.99-1.01) | 0.98 (0.97-0.99) |
| 3^rd^ (highest) | 41 271 (73.1%) | 55.5 | 0.74 (0.74-0.75) | 0.94 (0.93-0.95) | 0.93 (0.91-0.94) |
| Abbreviations: SHR, subdistribution hazard ratio. 95% confidence intervals in parenthesis. SHRs estimated by Fine-Gray regression with all-cause death as competing event. Adjusted analyses included the following variables: age, gender, calendar year of AF diagnosis, stroke, and bleeding risk factors (hypertension, heart failure, coronary artery disease, diabetes, prior stroke or transient ischemic attack, abnormal liver function, abnormal kidney function, prior bleeding episodes, concomitant use of nonsteroidal anti-inflammatory drugs or antiplatelets), dementia, cancer, alcohol use disorder, psychiatric disorders, income, and educational attainment. | | | | | |
