# Supplementary material for: Rural-urban differences in the initiation of oral anticoagulant therapy in patients with incident atrial fibrillation: A Finnish nationwide cohort study
Source: PLoS One. 2022 Oct 31;17(10):e0276612. doi: 10.1371/journal.pone.0276612 (PMC9621410; doi:10.1371/journal.pone.0276612)
Supplement: S3 Table — (DOCX) [file pone.0276612.s003.docx]

**S3 Table.** Incidence rate ratios and adjusted rate difference of OAC initiation during follow-up estimated with Poisson regression

|  | **Unadjusted IRR** | **Adjusted IRR** | **Adjusted rate difference (event/100 patient years)** |
| --- | --- | --- | --- |
| **Residence** | | |  |
| Rural | (Reference) | (Reference) | (Reference) |
| Urban | 0.89 (0.88-0.90) | 0.94 (0.93-0.95) | -3.4 (-3.9 - -2.8) |
| **Urbanization degree tertiles** | | |  |
| 1^st^ (lowest) | (Reference) | (Reference) | (Reference) |
| 2^nd^ | 0.90 (0.89-0.91) | 0.96 (0.95-0.97) | -2.2 (-2.8 - -1.7) |
| 3^rd^ (highest) | 0.83 (0.82-0.84) | 0.91 (0.90-0.93) | -5.0 (-5.6 - -3.9) |
| Abbreviations: IRR, incidence rate ratio. 95% confidence intervals in parenthesis. IRRs and rate differences estimated by Poisson regression. Adjusted analyses included the following variables: age, gender, calendar year of AF diagnosis, stroke, and bleeding risk factors (hypertension, heart failure, coronary artery disease, diabetes, prior stroke or transient ischemic attack, abnormal liver function, abnormal kidney function, prior bleeding episodes, concomitant use of nonsteroidal anti-inflammatory drugs or antiplatelets), dementia, cancer, alcohol use disorder, psychiatric disorders, income, and educational attainment. | | | |
