# Supplementary material for: Rural-urban differences in the initiation of oral anticoagulant therapy in patients with incident atrial fibrillation: A Finnish nationwide cohort study
Source: PLoS One. 2022 Oct 31;17(10):e0276612. doi: 10.1371/journal.pone.0276612 (PMC9621410; doi:10.1371/journal.pone.0276612)
Supplement: S4 Table — (DOCX) [file pone.0276612.s004.docx]

**Supplementary Table 4.** Hazard ratios of OAC initiation during follow-up estimated with Cox regression with death considered as informative censoring

|  | **Adjusted HR** |
| --- | --- |
| **Residence** | |
| Rural | (Reference)  0.95 (0.95-0.96) |
| Urban |  |
| **Urbanization degree tertiles** | |
| 1^st^ (lowest) | (Reference)  0.97 (0.96-0.98)  0.95 (0.94-0.96) |
| 2^nd^ |  |
| 3^rd^ (highest) |  |
| Abbreviations: HR, hazard ratio. 95% confidence intervals in parenthesis. HRs estimated by Cox regression with death treated as informative censoring using inverse probability of weighted censoring and adjusted for the following variables: age, gender, calendar year of AF diagnosis, stroke, and bleeding risk factors (hypertension, heart failure, coronary artery disease, diabetes, prior stroke or transient ischemic attack, abnormal liver function, abnormal kidney function, prior bleeding episodes, concomitant use of nonsteroidal anti-inflammatory drugs or antiplatelets), dementia, cancer, alcohol use disorder, psychiatric disorders, income, and educational attainment. | |
