# Supplementary material for: Rural-urban differences in the initiation of oral anticoagulant therapy in patients with incident atrial fibrillation: A Finnish nationwide cohort study
Source: PLoS One. 2022 Oct 31;17(10):e0276612. doi: 10.1371/journal.pone.0276612 (PMC9621410; doi:10.1371/journal.pone.0276612)
Supplement: S1 Fig — (PDF) [file pone.0276612.s005.pdf]

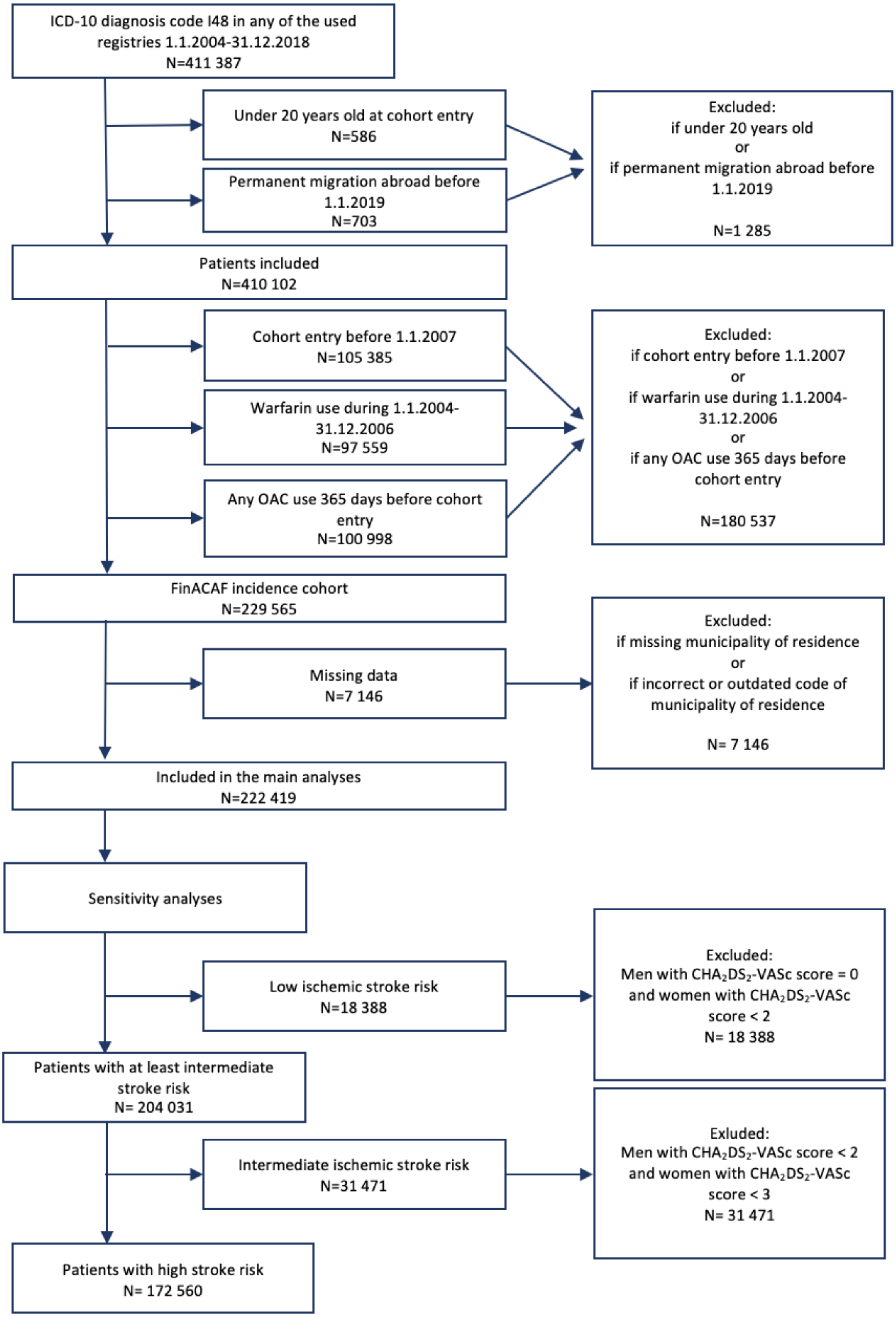
ICD-10 diagnosis code I48 in any of the used registries 1.1.2004-31.12.2018
N=411 387
Under 20 years old at cohort entry
N=586
Permanent migration abroad before 1.1.2019
N=703
Excluded:
if under 20 years old
or
if permanent migration abroad before 1.1.2019
N=1 285
Patients included
N=410 102
Cohort entry before 1.1.2007
N=105 385
Warfarin use during 1.1.2004-31.12.2006
N=97 559
Any OAC use 365 days before cohort entry
N=100 998
Excluded:
if cohort entry before 1.1.2007
or
if warfarin use during 1.1.2004-31.12.2006
or
if any OAC use 365 days before cohort entry
N=180 537
FinACAF incidence cohort
N=229 565
Missing data
N=7 146
Excluded:
if missing municipality of residence
or
if incorrect or outdated code of municipality of residence
N= 7 146
Included in the main analyses
N=222 419
Sensitivity analyses
Low ischemic stroke risk
N=18 388
Excluded:
Men with CHA_2DS_2-VASc score = 0 and women with CHA_2DS_2-VASc score < 2
N= 18 388
Patients with at least intermediate stroke risk
N= 204 031
Intermediate ischemic stroke risk
N=31 471
Exluded:
Men with CHA_2DS_2-VASc score < 2 and women with CHA_2DS_2-VASc score < 3
N= 31 471
Patients with high stroke risk
N= 172 560
